# A Chitin-Like Component on Sclerotic Cells of *Fonsecaea pedrosoi* Inhibits Dectin-1-Mediated Murine Th17 Development by Masking β-Glucans

**DOI:** 10.1371/journal.pone.0114113

**Published:** 2014-12-09

**Authors:** Bilin Dong, Dongsheng Li, Ruoyu Li, Sharon C.-A. Chen, Weihuang Liu, Wei Liu, Liuqing Chen, Yao Chen, Xu Zhang, Zhongsheng Tong, Yun Xia, Ping Xia, Yan Wang, Yiqun Duan

**Affiliations:** 1 Center for Infectious Skin Diseases, Department of Dermatology, No. 1 Hospital of Wuhan, Wuhan, China; 2 Research Center for Medical Mycology, Peking University, Beijing, China; 3 Center for Infectious Diseases and Microbiology, Laboratory Services, ICPMR–Pathology West, Westmead Hospital, University of Sydney, Westmead, Australia; 4 Medical Research Center of Wuhan University, Wuhan, China; 5 Institute of Hydrobiology, Chinese Academy of Sciences, Wuhan, China; Leibniz Institute for Natural Products Research and Infection Biology- Hans Knoell Institute, Germany

## Abstract

*Fonsecaea pedrosoi* (*F. pedrosoi*), a major agent of chromoblastomycosis, has been shown to be recognized primarily by C-type lectin receptors (CLRs) in a murine model of chromoblastomycosis. Specifically, the β-glucan receptor, Dectin-1, mediates Th17 development and consequent recruitment of neutrophils, and is evidenced to have the capacity to bind to saprophytic hyphae of *F. pedrosoi* in vitro. However, when embedded in tissue, most etiological agents of chromoblastomycosis including *F. pedrosoi* will transform into the sclerotic cells, which are linked to the greatest survival of melanized fungi in tissue. In this study, using immunocompetent and athymic (nu/nu) murine models infected subcutaneously or intraperitoneally with *F. pedrosoi*, we demonstrated that T lymphocytes play an active role in the resolution of localized footpad infection, and there existed a significantly decreased expression of Th17-defining transcription factor Rorγt and inefficient recruitment of neutrophils in chronically infected spleen where the inoculated mycelium of *F. pedrosoi* transformed into the sclerotic cells. We also found that Dectin-1-expressing histocytes and neutrophils participated in the enclosure of transformed sclerotic cells in the infectious foci. Furthermore, we induced the formation of sclerotic cells in vitro, and evidenced a significantly decreased binding capacity of human or murine-derived Dectin-1 to the induced sclerotic cells in comparison with the saprophytic mycelial forms. Our analysis of β-glucans-masking components revealed that it is a chitin-like component, but not the mannose moiety on the sclerotic cells, that interferes with the binding of β-glucans by human or murine Dectin-1. Notably, we demonstrated that although Dectin-1 contributed to the development of IL-17A-producing CD3+CD4+ murine splenocytes upon in vitro-stimulation by saprophytic *F. pedrosoi*, the masking effect of chitin components partly inhibited Dectin-1-mediated Th17 development upon in vitro-stimulation by induced sclerotic cells. Therefore, these findings extend our understanding of the chronicity of chromoblastomycosis.

## Introduction

Chromoblastomycosis is a chronic cutaneous and subcutaneous mycosis caused by melanized fungi, of which *Fonsecaea pedrosoi* is considered as the most common agent [1], [2], [3]. Although systemic invasion is rare, localized chromoblastomycosis is often progressive and is associated with clinical complications including lymphoedema and malignant transformation of such long-standing lesions [2], [4], [5]. Furthermore, there is often a poor response to oral antifungal drugs, and most attempts at treatment have only a modest success rate [2], [3], [6].

It is known that T helper (Th) cells play a crucial role in adaptive immunity against fungi through the secretion of distinct cytokine profiles [7], [8]. Specifically, absence of CD4+ T cells impairs host defense against *F. pedrosoi* infection in experimental murine models [6], [9]. Furthermore, notable features of patients with chromoblastomycosis include increased IL-10 and low levels of IFN-γ [10]. More recently, IL-17 secreted by a subset of Th cells, called IL-17-producing Th (Th17) cells, has been demonstrated to recruit neutrophils required for anti-fungal response in a manner dependent on IL-17/IL-8 axis [11]–[13]. Both mice and humans with impaired IL-17 production or IL-17R deficiency are prone to infections with invasive fungal pathogens including *Candida* and *Aspergillus* [8], [14]–[16]. However, the Th17 response in chromoblastomycosis remains to be elucidated.

In addition, studies have shown that *F. pedrosoi* is recognized primarily by C-type lectin receptors (CLRs) in a murine model of chromoblastomycosis, resulting in the defective induction of pro-inflammatory cytokines [3]. Dectin-1, a non-TLR pattern-recognition receptor (PRR), shows features of a kind of type II transmembrane receptor that contains a single C-type lectin domain (CTLD) in the extracellular region and an immunoreceptor tyrosine-based activation (ITAM)-like motif within its intracellular tail [17]–[19]. The receptor is widely expressed on innate immune cells including dendritic cells, monocytes/macrophages and neutrophils [17]–[19]. Upon specific recognition of fungal β-1,3-glucans, its ITAM-like motif initiates Syk/CARD9-mediated signaling pathway, and stimulates the secretion of Th17-inducing cytokines [20], [21]. Recombinant human-derived Dectin-1 has been shown to bind to saprophytic forms of *F. pedrosoi* [3].

However, when embedded in tissue, most etiological agents of chromoblastomycosis including *F. pedrosoi* transforms into the parasitic phase-the sclerotic cells, which are extremely resistant to phagocytosis and destruction by host cells, and are linked to the greatest survival of melanized fungi in tissue [22], [23]. Notably, Garnter, *et al* suggested that the exposure of β-1,3-glucan on yeast cell wall is mainly restricted to the bud and birth scars [18]. Given that formation of the sclerotic cell is associated with a thickened cell wall, and in particular, the disappearance of bud scars [23], [24], we hypothesize that the ability of Dectin-1 to bind to *F. pedrosoi* will be attenuated with the conversion of saprophytic phase into sclerotic cells, and that Dectin-1-mediated Th17 development will be inhibited.

To address the above hypothesis, we demonstrated in the present work that in the BALB/c mice with chronic *F. pedrosoi* infection, there was a suppressed Th17 development with decreased neutrophil recruitment. Of note, the results of in vitro experiments showed that it is the chitin moiety on the cell wall of *F. pedrosoi* sclerotic cells that interferes with the immune recognition of β-glucans by human/murine-derived Dectin-1, which partly inhibits the development of Th17 cells.

## Materials and Methods

### Ethics Statement

Animal experiments in this study were performed in accordance with the recommendations in the Guide for the Care and Use of Laboratory Animals of the National Institutes of Health. Our study protocol was approved by the Institutional Animal Care and Use Committee of No. 1 Hospital of Wuhan (project license number: WHB201404016). To minimize suffering, mice were anaesthetized prior to sacrifice.

Fungal strain and five archived paraffin-embedded tissue sections from the patients with known chromoblastomycosis were provided by the Department of Dermatology, No. 1 Hospital of Wuhan, and were used without any linkage of patient data. The use of these samples was approved by the Institutional Review Board, No. 1 Hospital of Wuhan (project license number: S038).

### Source of mice

Immunocompetent and athymic (nu/nu) BALB/c male mice (SPF, 6–8 wk old) were purchased from the Animal Laboratory Center, Wuhan University, and maintained in special pathogen-free conditions.

### Fungal strain, media, and growth conditions

*F. pedrosoi* strain (WH10-002) (Genbank no: GQ420654.1) was obtained from a patient with chromoblastomycosis in 2009. The strain was cultivated on Potato dextrose agar (PDA, DIFCO laboratories) supplemented with chloramphenicol at 50 µg/ml at 28°C, and was periodically transferred at 60-day intervals for preservation.

To induce the formation of saprophytic mycelium and conidia, the stock culture was incubated in Potato dextrose broth (PDB, DIFCO laboratories) pH 6.0, at 28°C for 15 days, under shaking.

To induce the formation of sclerotic cells, 15-day-old cultures grown in PDB were inoculated into synthetic basal medium (ATCC medium 830), pH 6.0, with the following composition (g/l): MgSO_4_, 0.1; NH_4_NO_3_, 1.5; KH_2_PO_4_, 1.8; Biotin, 5×10^−5^; thiamine-HCl, 1.0×10^−4^; Glycerol, 6.5, as previously described [24]–[26]. In addition, platelet-activating factor was added at a final concentration of 10^−6^ M. After incubation with gentle shaking (160 rpm) at 35.5°C for 50 days, the formation of sclerotic cells was confirmed by Scanning and Transmission Electronic Microscopy (S-4800; HT-7700, Hitachi).

### In vivo infection with saprophytic *F. pedrosoi*

To prepare fungal inocula, 15-day-old cultures in PDB were vortexed thoroughly to detach the conidia or conidiogenous cells from the mycelia. Afterwards, the upper part of fungal suspensions were passed through the 50-mL sterile syringe several times and then through a nylon filter (200 mesh), where the mycelial masses were retained. The filtrates containing conidia and solitary hyphae were washed and concentrated 3× in PBS by centrifugation at 6000 rpm for 5 min. The inoculum volumes were adjusted to a final concentration of 5×10^9^/mL with a Neubauer chamber.

Ten minutes prior to infection, animals were anaesthetized intraperitoneally (i.p.) with 0.4 µl of Anasedan and 0.35 ml of Dopalen per kg body weight [23]. For the immunocompetent and nu/nu BALB/c mice, 50 µl of inocula were injected subcutaneously (s.c.) into the footpad. For the remaining group of immunocompetent BALB/c mice, 100 µl of inocula were inoculated i.p. into the abdomen using a 25×8/21G1 needle. Mice were monitored weekly for up to 50 days post inoculation for clinical features of both local and systemic infection. Histopathological examination of footpad and spleen tissue was done on days 7, 14, 21, 36 and 50 day after inoculation. The mice inoculated s.c. or i.p. with normal saline were set as control.

### Detection of Myeloid peroxidase (MPO) and Dectin-1 via immunohistochemistry

Each of the three groups of mice studied were divided into 6 subgroups (each subgroup, n = 5) according to the time point of examination as above, in which the control group was included. Immunohistochemistry studies were also performed for the formalin-fixed, paraffin-imbedded tissue specimens of patients (n = 5) with chromoblastomycosis.

To detect MPO and Dectin-1 distribution in the infected footpad or spleen at the above-indicated time points, anti-MPO at a dilution of 1∶160 (R&D systems, AF3667), and anti-Dectin-1 at a dilution of 1∶200 (R&D systems, MAB17561) were used. HRP-conjugated secondary antibodies were purchased from Santa cruz biotechnologies. The immunohistochemistry staining was performed as previously described [27].

### Detection of MPO and Rorγt via Western blotting

To prepare protein extracts for western blotting, infected footpads and spleen tissues harvested from each group of mice were homogenized on ice in RIPA buffer (Sigma-aldrich, R0278) added with 1× protease inhibitor cocktails (Thermo, 78410) and 1 mM phenylmethylsuphonylfluoride (PMSF) (Sigma-aldrich, P7626). Total protein was quantified via Bicinchoninic Acid Assay (Pierce), denatured with 2× laemmli buffer (Sigma-aldrich, 38733) and heated to 95°C for 5 min. 20 µg total protein was loaded onto each well of a 7.5% polyacrylamide gel (BioRad), separated via electrophoresis, and transferred to PVDF membrane (Sigma-aldrich, P2438). Blots were stained accordingly with anti-Rorγt (Ebioscience, 14-6981-82), anti-MPO (R&D systems), and anti-β-actin (Cell signaling, 4967), and then with HRP-conjugated secondary antibodies (Santa cruz biotechnologies). All blots were developed using the GE Healthcare ECL Western Blotting Detection Reagent (Amersham). The protein transfer zone was shown on photosensitive film (Hyperfilm, GE Healthcare) by rapidly developing and fixing in the darkroom, and the optical density was analyzed with AlphaView analysis software.

### Treatment of in vitro-induced sclerotic cells with hydrogen peroxide

Cultures containing sclerotic cells were respectively treated with 1%, 3%, 10% and 30% hydrogen peroxide (H_2_O_2_) for 30 min, and any destruction of the fungal cell wall was detected by laser confocal microscopy and transmission electronic microscopy (HT-7700, Hitachi).

### Dectin-1 binding assay

The saprophytic cultures in PDB and induced cultures containing sclerotic cells in ATCC medium 830 were respectively pretreated to detach the mycelia, and then filtrated according to methods mentioned above. Culture filtrates were then washed 3× in PBS, and adjusted to a concentration of 1.0×10^7^ CFU/mL in PBS containing 3% bovine serum albumin (BSA). A total of 100 µL fungal cell suspension was incubated with human- or murine-derived recombinant Dectin-1 (10 µg/mL, 1859-DC-050; 1756-DC-050, R&D systems) at 37°C for 60 min. After washed in PBS, 100 µL fungal cell suspension (1.0×10^7^ CFU/mL) was incubated with 10 µL PE-conjugated anti-human or PE-conjugated anti-murine Dectin-1 (FAB1859P; FAB17561P, R&D systems) at 4°C for another 45 min. β-1,3-D-glucanase from *Helix pomatia* (9044-93-3, Sigma-aldrich) was used to identify whether the specific recognition and binding site of human or murine Dectin-1 was the β-1,3-D-glucan moiety of fungal cell wall. Briefly, fungal cell suspension was pre-incubated with β-1,3-D-glucanase (10 U/mL) at 37°C for 60 min and washed 3× in PBS prior to indirect immunofluorescence staining. The fungal cells incubated only with PE-conjugated anti-human or only with anti-murine Dectin-1 were set as fluorescence controls. Dectin-1 binding was detected by confocal laser scanning microscopy and flow cytometry assay.

### MR binding assay

To detect the binding of human or murine mannose receptor (MR) to sclerotic cells, the prepared sclerotic cell cultures in PBS (1.0×10^7^ CFU/mL, 100 µL) were incubated with recombinant human or murine MR/CD206 (10 µg/mL, 2534-MR-050; 2535-MM-050, R&D systems) at 37°C for 60 min. After washed in PBS, 100 µL of fungal cell suspension (1.0×10^7^ CFU/mL) were incubated with 10 µL Fluor 488-conjugated anti-human or anti-murine MR (FAB25342G; FAB2535G, R&D systems) at 4°C for another 45 min. The sclerotic cells incubated only with Fluor 488-conjugated anti-human or only with anti-murine MR were set as fluorescence controls. The binding of human or murine mannose receptor to the induced sclerotic cells was detected by laser confocal micoroscopy.

### Chitinase treatment and WGA binding assay

For chitinase treatment, the prepared saprophytic and induced cultures (1.0×10^7^ CFU/mL, 100 µL) were incubated with 2 mL PBS containing 5 U chitinase (C6137, Sigma-aldrich) at 37°C overnight.

In addition, FITC-conjugated Wheat Germ Agglutinin (WGA) was used according to the manufacturer's protocol (L4895, Sigma-aldrich) to detect the distribution of chitin moiety on fungal cells. Briefly, the prepared fungal suspensions (1.0×10^7^ CFU/mL, 100 µL) with or without chitinase treatment were incubated with FITC-WGA (1 mg/mL) at 4°C for 90 min. After washed in PBS, the binding of WGA to saprophytic or induced cultures was respectively detected by laser confocal micoroscopy and flow cytometry assay. The fungal cells (1.0×10^7^ CFU/mL) without any treatment were set as self-fluorescence control. SEM (S-4800, Hitachi) was used to observe the cell wall ultrastructure of saprophytic hyphae or induced sclerotic cells before and after chitinase digestion.

To determine the masking effect of chitin moiety on the β-glucans of sclerotic cells induced *in vitro*, the binding of human or murine Dectin-1 to the sclerotic cells was detected in the presence or absence of chitinase treatment.

### Preparations of splenocytes from BALB/c murine inoculated i.p. with *F. pedrosoi*

The spleens were aseptically removed from the BALB/c mice i.p. inoculated with saprophytic *F. pedrosoi* on day 7 and 50 post-inoculation, and then meshed through a cell sieve. After lysis of red blood cells, the spleen suspensions were washed 3× in PBS, and adjusted to a concentration of 1.0×10^7^/mL. The percentage of viable cells obtained was more than 95% as determined by trypan blue staining. The splenocytes from the group of BALB/c mice inoculated i.p. with N.S were set as controls.

### Immunofluorescence staining for Dectin-1 and CD11c

The prepared splenocytes (1.0×10^7^/mL, 100 µL) from the groups BALB/c mice mentioned above were incubated with 10 µL PE-conjugated anti-Dectin-1 (FAB17561P, R&D systems), and 2 µL FITC-conjugated anti-CD11c (11-0114-81, Ebioscience) at 4°C for 45 min. Afterwards, the suspensions were washed 3× in PBS, and the fluorescence was measured by flow cytometry. The groups of splenocytes incubated with FITC-conjugated Armenian Hamster IgG or PE-conjugated rat IgG2A were set as isotype controls.

### Cell culture, Dectin-1 blockage, and stimulation with *F. pedrosoi*

Splenocytes from non-infected, i.p.-infected, and s.c.-infected BALB/c mice at 50 days post-inoculation (each group, n = 5) were respectively adjusted to a concentration of 5.0×10^5^/mL in RPMI 1640, and incubated with 5 µg/mL anti-mouse Dectin-1 blocking mAb (FAB17561, R&D systems) at 37°C for 90 min. The performance of abrogation was detected by flow cytometry using PE-conjugated anti-Dectin-1 (FAB17561P, R&D systems).

Prior to stimulation, the majority of hyphal filaments in saprophytic and induced cultures of *F. pedrosoi* were removed as described above, and the fungal suspensions were adjusted to a concentration of 1.5×10^6^/mL in RPMI 1640. For inhibition of morphological changes in the presence of serum, fungal cells were stored in PBS at 4°C for at least 45 days before stimulation. Thereafter, the splenocytes with or without Dectin-1 blocking mAb pretreatment were plated in 24-well plates (5.0×10^5^/well), and then were stimulated with saprophytic *F. pedrosoi* or in vitro-induced sclerotic cells in the presence or absence of chitinase pretreatment at a ratio of 1∶3 in a volume of 2 mL RPMI-1640 medium containing 10% heat-inactivated FCS and rmIL-2 (100 U/mL). The mixed cultures were incubated in a 5% CO_2_ incubator at 37°C for 7 days, and IL-17A-producing Th cells were analyzed by flow cytometry.

### Immunofluorescence staining for CD3, CD4 and intracellular IL-17A

The immunofluorescence staining was performed according to the protocols provided by eBioscience with minor modifications. Briefly, untreated or *F. pedrosoi*-stimulated murine splenocytes in the absence or presence of 5 µg/mL Dectin-1 blocking mAb were cultured with 1× Cell Stimulation Cocktail (500×) (00-4970, eBioscience) and 1× Protein Transport Inhibitor Cocktail (500×) (00-4980, eBioscience) for 12 hours. After washed 3× in PBS containing 5% BSA, the cell pellets were incubated with FITC-CD3 (11-0032-82, eBioscience) and PE-Cy5-CD4 (15-0041-81, eBioscience) at 4°C for 30 min in the dark. For intracellular staining, the splenocytes were firstly fixed and permeabilized using IC fixation buffer (00-8222, eBioscience) and permeabilization buffer (00-8333, eBioscience), and then incubated with PE-IL-17A (12-7177, eBioscience) or with intracellular PE-Rat IgG2a (12-4321-41, eBioscience, isotype) at 4°C for another 30 min in the dark. The staining was detected by flow cytometry assay.

### Laser confocal microscopy and flow cytometry assay

The two-photon Laser Scanning Microscope (LSM710, Zeiss) and Flow Cytometer (Epics Altra II, Beckman Coulter, USA) were used for immunofluorescence staining studies. The excitation and emission wavelengths of 488 and 525 nm were used for FITC or Fluor-488 assay, 488 and 585 nm for PE-assay, and 488 and 695 nm for PE-Cy5 assay.

### Statistical analysis

The results of all experiments in this study were presented as mean ± SEM. Statistical significance was determined using Univariate ANOVA in SPSS-13.0 followed by post-hoc analysis including LSD-t test. A *P* value of <0.05 was considered significant.

## Results

### Immunocompetent BALB/c mice inoculated s.c. with *F. pedrosoi* developed to be self-healing

**Figure 1:**
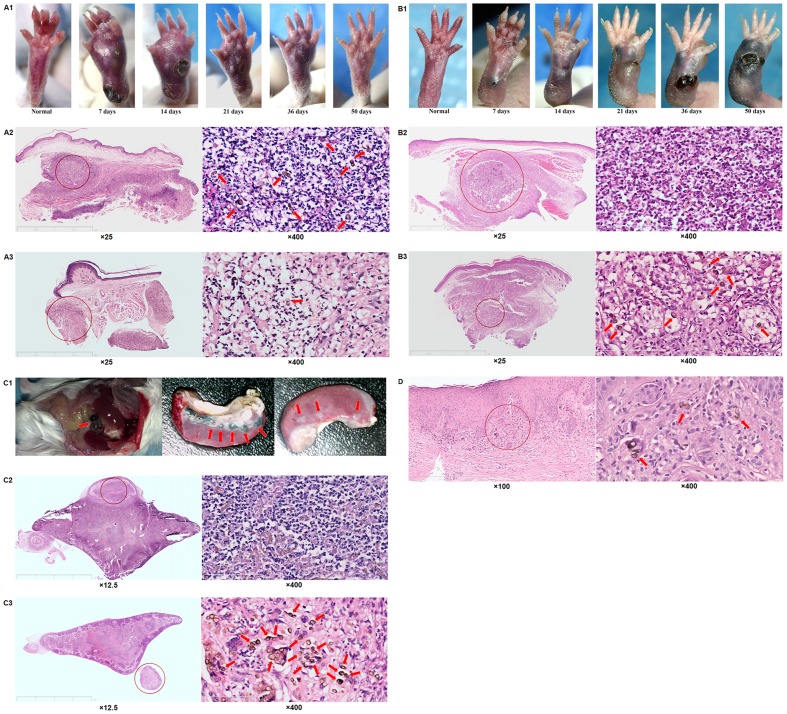
An inefficient recruitment of neutrophils was observed in chronically infected foci due to *F. pedrosoi*. (A1–A3) Immunocompetent and athymic (nu/nu) (B1–B3) BALB/c mice were respectively inoculated s.c. with saprophytic *F. pedrosoi*. (A1 and B1) Images of normal and infected footpads were taken at days 7, 14, 21, 36 and 50 post-inoculation. Infected footpads at days 7 (A2 and B2) and 36 (A3 and B3) were respectively stained for H&E to analyze histological characteristics. (C1–C3) Immunocompetent BALB/c mice were inoculated i.p. with saprophytic *F. pedrosoi*. (C1) Images of infected foci were taken at days 15(left) and 50 (middle and right) post-inoculation. Infected spleens at days 7 (C2) and 50 (C3) were respectively stained for H&E to analyze histological characteristics. (D1–D2) Tissue sections of the patient with chromoblstomycosis over 10 years were stained for H&E to analyze histological characteristics. (A–D) The transformed sclerotic cells were indicated by red arrows.

For this group of immunocompetent BALB/c mice, swollen footpads occurred and developed with ulcers and necrosis as a result of transepithelial liberation of abscess from day 7 till 18–20 post-inoculation (Fig. 1-A1). Clinical improvement manifest by a decreased volume of swollen footpad was observed from day 21 to 36 post-inoculation, and clinical cure was observed after 50 days post-inoculation (Fig. 1-A1). Histological sections showed a persistent recruitment of neutrophils into the infected site from about day 7 to 36 post-inoculation, and the formation of micro-abscess in which the sclerotic cells of *F. pedrosoi* were surrounded and eliminated by neutrophils (Fig. 1-A2 and A3).

### Athymic (nu/nu) BALB/c mice infected s.c. with *F. pedrosoi* were prone to be chronically infected

In contrast to the self-healing pattern of immunocompetent BALB/c mice infected subcutaneously, the footpad abscess seen in nu/nu BALB/c mice began to form at 5–7 days post-inoculation, and gradually disseminated to the whole footpad from the inoculation site at 50 days post-inoculation (Fig. 1-B1). In addition, transepithelial liberation of abscess was not observed until 30–32 days post-inoculation, and the volume of infected footpads increased within the 50-day observation period (Fig. 1-B1). Histological examination of the sections showed that although there was an early influx of neutrophils into the infectious foci where dematiaceous hyphae were found at 7 days post-inoculation, a significantly decreased infiltration of neutrophils was observed in foci where the inoculated *F. pedrosoi* transformed into the sclerotic cells at 50 days post-inoculation (Fig. 1-B2 and B3).

### Immunocompetent BALB/c mice inoculated i.p. with *F. pedrosoi* were prone to be chronically infected

For the group of immunocompetent mice inoculated i.p. with *F. pedrosoi*, multiple small black or dark-brown nodules on the abdomen began to form after 7–10 days post-inoculation, and then disseminated to the peritoneal serosa and to the stomach, bowel, liver, and spleen (Fig. 1-C1). The spleen was affected at 12–15 days post-inoculation, and multi-nodular lesions were seen within the 50-day period (Fig. 1-C1). Histological sections showed well-encapsulated abscesses in the infected spleen where dematiaceous hyphae were surrounded by numerous neutrophils at 7–10 days post-inoculation (Fig. 1-C2). However, the number of infiltrated neutrophils in the infectious foci decreased significantly with the transformation of *F. pedrosoi* into the parasitic sclerotic cells within 50 days post-inoculation (Fig. 1-C3).

### Histological characteristics of patients with chromoblastomycosis

Histological sections of patients with long-standing (>10 years) chromoblastomycosis showed that the infiltration of neutrophils was not obvious in the infectious foci where the parasitic sclerotic cells were enclosed by poly-nuclear giant cells (Fig. 1D).

### Decreased expressions of MPO and Rorγt were observed in chronically infected, but not in self-healing murine models

#### Immunohistochemistry staining

**Figure 2:**
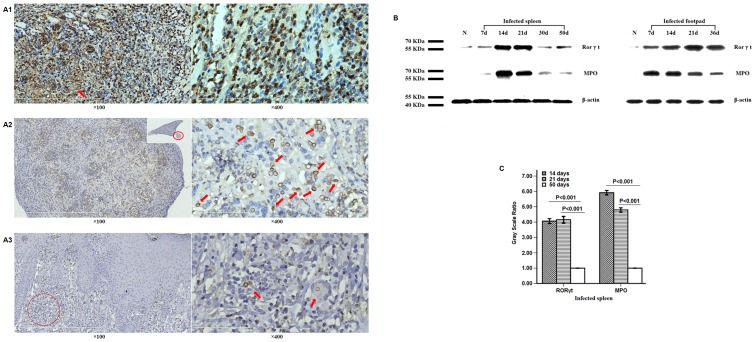
Decreased expressions of Rorγt and neutrophil MPO in chronically infected foci due to *F. pedrosoi*. (A1–A3) Photographs of immunohistostaining for MPO in the foci of self-healing (A1) and chronically infected murine models (A2), and the patient with chromoblastomycosis (A3). The hyphal elements (A1) or sclerotic cells (A2 and A3) of *F. pedrosoi* were indicated by red arrows. (B and C) Western blotting assay was used to detect the expressions of Rorγt and MPO in the infected spleens or footpads of BALB/c mice at the indicated time point after inoculation. Data are shown as mean ±±SEM from one representative experiment, with 5 mice per time point and significance tested using LSD-t test.

For the groups of immunocompetent BALB/c mice inoculated s.c. or i.p. with *F. pedrosoi*, the expression of MPO was observed in the infiltrated neutrophils, but not in the histocyte-like cells in the infectious foci at 5–7 days post-inoculation (Fig. 2-A1). With significantly decreased recruitment of neutrophils, histological sections showed that the expression of MPO was not obvious in the infected spleen at 50 days post-inoculation or in the infectious foci of patients with long-standing chromoblastomycosis (Fig. 2-A2 and A3).

#### Western blotting analysis

For the chronically infected murine models inoculated i.p. with *F. pedrosoi*, the expression levels of Rorγt, the nuclear transcript which is essential for Th17 development, and MPO in the infected spleens at 50 days post-inoculation were respectively lower than those at 14 days or 21 days post-inoculation (LSD-t test, p<0.01) (Fig. 2B and 2C).

However, for the self-healing murine models inoculated s.c. with *F. pedroso*i, persistent expressions of MPO and Rorγt were observed for as long as 36 days after inoculation when the infected footpads had a significant improvement (Fig. 1-A1 and Fig. 2B).

### In vitro-transformed sclerotic cells were damaged by hydrogen peroxide

**Figure 3:**
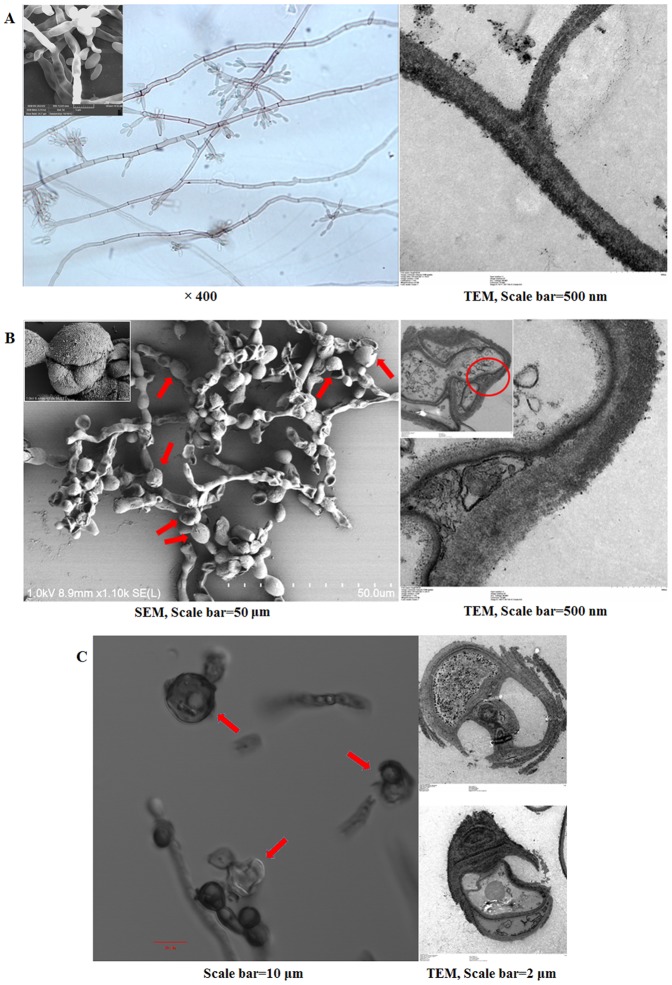
In vitro-transformed sclerotic cells with multi-planate division were damaged by hydrogen peroxide. (A and B) SEM and TEM as well as optical microscope were used to characterize the morphology of saprophytic *F. pedrosoi* (A) and transformed sclerotic cells in ATCC medium 830 (B), as indicated by the red arrows. TEM, scale bar  = 500 nm; SEM, scale bar  = 50 µm. (C) Confocal microscope and TEM were used to observe the integrity of transformed sclerotic cells after treatment with 1% hydrogen peroxide. The destroyed sclerotic cells were indicated by red arrows. Confocal, scale bar  = 10 µm; TEM, scale bar  = 2 µm.

We observed the morphological changes of in vitro-transformed sclerotic cells in ATCC medium 830 added with PAF, including an increased cell wall and the formation of multi-planate division in comparison with the structure of saprophytic *F. pedrosoi* (Fig. 3A and 3B).

When treated with hydrogen peroxide at a concentration of 1% or higher for 30 min, the destroyed structure of in vitro-induced sclerotic cells including the broken septum and imperfect cell wall was observed by confocal microscope and TEM (Fig. 3C).

### Dectin-1-positive inflammatory cells were recruited into the infectious foci

**Figure 4:**
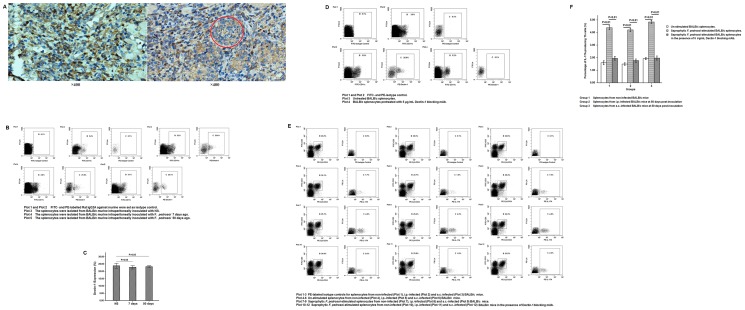
Dectin-1 partly mediated Th17 development in murine splenocytes upon in vitro stimulation with saprophytic *F. pedrosoi*. (A) Photographs of immunohistostaining for Dectin-1 in the infected foci due to *F. pedrosoi*. The sclerotic cell in the foci was indicated by the red circle. (B and C) The percentage of Dectin-1+CD11c+ cells in the CD11c-expressing splenocytes was measured by PE-anti-murine Dectin-1 mAb and FITC-anti-murine CD11c mAb using flow cytometry at indicated time points after inoculation. (B) Gate B represents the percentage of CD11c+ cells in the whole splenocytes from mice, and Gate C represents the percentage of Dectin-1+CD11c+ cells in the CD11c-expressing splenocytes. Gate B is related to Gate C. (C) Data are shown as mean ±±SEM from one representative experiment performed in triplicates, with 5 mice per indicated time point and significance tested using LSD-t test. (D) The blockage of Dectin-1 expression on CD11c+ splenocytes in the presence of 5 µg/mL Dectin-1 blocking mAb was detected using PE-anti-murine Dectin-1 mAb and FITC-anti-murine CD11c mAb. The groups of splenocytes incubated with FITC-conjugated Armenian Hamster IgG or PE-conjugated rat IgG2A were set as isotype controls. (E) The percentage of IL-17A-expressing Th cells in CD3+CD4+ splenocytes was detected by FITC-anti-murine CD3, PE-Cy5-anti-murine CD4 and PE-anti-murine IL-17A using flow cytometry. The splenocytes stained with FITC-CD3 and PE-Cy5-CD4, and then with intracellular PE-Rat IgG2a were set as isotype controls. (F) Data are shown as mean ±±SEM from one representative experiment performed in triplicates, with 5 mice in each group (non-infected, i.p-infected and s.c.-infected at 50 days post-inoculation) and significance tested using LSD-t test.

Immunohistochemistry staining showed that Dectin-1-positive neutrophils and histocyte-like cells were recruited into the infectious foci, and contributed to the enclosure of sclerotic cell (Fig. 4A).

### The percentage of Dectin-1+CD11c+ splenocytes from BALB/c mice remained almost unchanged during an i.p. infection with *F. pedrosoi*

Flow cytometry assay showed that there was no significant difference for the percentage of Dectin-1+CD11c+ cells in CD11c-expressing splenocytes from BALB/c mice among the 7 days post-inoculation group, 50 days post-inoculation group, and NS inoculation group (LSD-t test, P>0.05) (Fig. 4B and 4C).

### Dectin-1 partly mediates Th17 development from murine splenocytes upon in vitro stimulation with saprophytic *F. pedrosoi*

In the present study, the expression of Dectin-1 on the CD11c + splenocytes was completely abrogated by 5 µg/mL anti-mouse Dectin-1mAb (Fig. 4D). The CD3+CD4+ double-positive splenocytes were recorded as Th cells (Fig. 4E). Flow cytometry assay showed that the percentage of IL-17A-producing Th cells in all the non-infected, i.p. infected, and s.c. infected groups increased significantly when stimulated in vitro by saprophytic *F. pedrosoi* in comparison with those in the un-stimulated groups, or those in the stimulated groups in the presence of 5 µg/mL Dectin-1 blocking mAb (LSD-t test, P<0.01) (Fig. 4E and 4F). But no significant variation was found among those in the three groups in-vitro stimulated by saprophytic *F. pedrosoi* (LSD-t test, P>0.05) (Fig. 4E and 4F).

### A decreased binding affinity of human- or murine-derived Dectin-1 for β-glucans was observed with the transformation of saprophytic *F. pedrosoi* into the sclerotic cells

**Figure 5:**
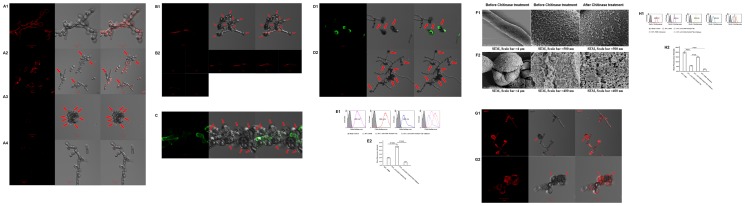
A chitin-like component masks the immunorecognition of β-glucans on sclerotic cells by Dectin-1. (A1–A4) The binding of murine-derived Dectin-1 to the saprophytic *F. pedrosoi* in PDB before (A1) and after (A4) β-glucanase treatment, or to the 30-day (A2) and 50-day (A3) induced cultures containing sclerotic cells in ATCC medium 830 was detected by PE-anti-murine Dectin-1 mAb using confocal microscope. (B1 and B2) The binding of human-derived Dectin-1 to the induced cultures containing sclerotic cells was detected by PE-anti-human Dectin-1 mAb using confocal microscope. (C) The binding of murine-derived mannose receptor (MR) to the induced cultures containing sclerotic cells and chlamydospores was detected by FITC-anti-murine MR using confocal microscope. (D) FITC-WGA was used to detect the distribution of chitin-like component on the induced cultures containing sclerotic cells before (D1) and after (D2) Chitinase treatment using confocal microscope. (E1 and E2) The binding capacity of FITC-WGA to the saprophytic *F. pedrosoi* or to the induced cultures before and after chitinase treatment was detected using flow cytometry assay, and represented as the Mean Fluorescence Intensity (MFI). The induced cultures without any treatment were set as blank control. (F1 and F2) SEM was used to observe the surface ultra-structure of saprophytic hypha (F1) and in vitro-transformed sclerotic cells (F2) in the presence or absence of chitinase pretreatment. SEM, Scale bar  = 4 µm (left) or 500 nm (middle and right). (G1 and G2) The binding of murine-derived Dectin-1 to the induced cultures containing sclerotic cells with chitinase pretreatment was detected by confocal microscope. (A-D, G) The transformed sclerotic cells and chlamydospores were indicated by red arrows. (H1 and H2) The binding capacity of murine-derived Dectin-1 to the saprophytic *F. pedrosoi* before and after β-glucanase treatment, or to the induced cultures before and after chitinase treatment was detected by indirect immunofluorescence assay using flow cytometer, and represented as the Mean Fluorescence Intensity (MFI). The fungal cells only incubated with PE-conjugated anti-murine Dectin-1 mAb were set as blank control. (E2 and H2) Data are shown as mean ± SEM from three individual experiments performed in triplicates, and significance tested using LSD-t test.

We observed that the binding site of human- or murine-derived Dectin-1 was mainly distributed to the hyphae and conidiospores of saprophytic *F. pedrosoi*, but not to the in vitro-transformed sclerotic cells and chlamydospores using confocal microscope (Figs. 5A and 5B). Flow cytometry assay also showed that the binding capacity of murine Dectin-1 to the saprophytic *F. pedrosoi* in PDB was significantly stronger than that to the induced cultures in ATCC medium 830 with PAF (LSD-t test, P<0.01) (Fig. 5H). After the treatment of β-1, 3-D-glucanase, a significantly decreased binding capacity of murine Dectin-1 to *F. pedrosoi* in PDB was observed (LSD-t test, P<0.01) (Figs. 5A and 5H).

### A chitin-like component, but not the mannose moiety, on the sclerotic cells attenuates the binding of Dectin-1 to β-1, 3-D-glucans

We observed that the binding site of human- or murine-derived mannose receptor (MR) was largely distributed over the surface of hyphae, but not over the transformed sclerotic cells or chlamydospores using confocal microscope (Fig. 5C). However, the binding of WGA was observed on the surface of a majority of transformed sclerotic cells, and the terminal part of vegetative hyphae (Fig. 5D). Flow cytometry further showed that the binding capacity of WGA to the induced cultures in ATCC medium 830 was significantly stronger than that to the saprophytic *F. pedrosoi* in PDB, or the induced cultures with chitinase pretreatment (LSD-t test, P<0.01) (Fig. 5E). SEM examination further showed that the surface structure of the induced sclerotic cells, but not the saprophytic hyphae, became looser when treated with chitinase (Fig. 5F).

In addition, the binding of human- or murine-derived Dectin-1 to the transformed sclerotic cells became obvious after chitinase treatment using confocal microscope. Flow cytometry assay also showed a significantly increased binding capacity of Dectin-1 to the induced cultures which were pretreated with chitinase in comparison with the untreated one (LSD-t test, P<0.01) (Fig. 5G and 5H).

### Chitin moieties partly inhibits Dectin-1-mediated Th17 development upon in vitro stimulation with induced sclerotic cells

**Figure 6:**
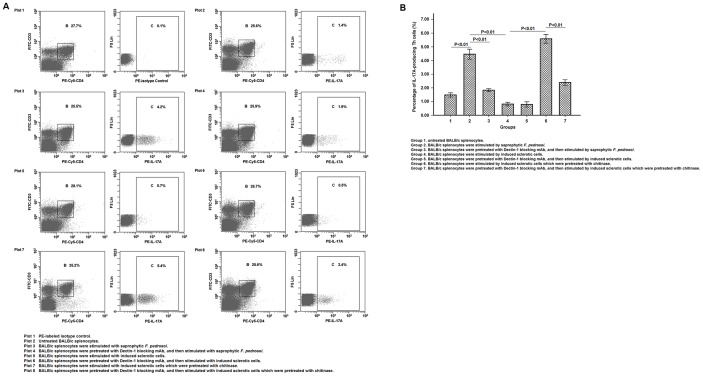
Chitin moieties on the sclerotic cells of *F. pedrosoi* partly suppressed Dectin-1-mediated Th17 development. (A) The percentage of IL-17A-expressing Th cells in CD3+CD4+ splenocytes was detected by FITC-anti-murine CD3, PE-Cy5-anti-murine CD4 and PE-anti-murine IL-17A using flow cytometry. The splenocytes stained with FITC-CD3 and PE-Cy5-CD4, and then with intracellular PE-Rat IgG2a were set as isotype controls. (B) Data are shown as mean ±±SEM from four individual experiments performed in triplicates, and significance tested using LSD-t test.

In the group stimulated by induced sclerotic cells, we observed a significantly decreased percentage of IL-17A-producing Th cells when compared with that in the group stimulated by saprophytic *F. pedrosoi* (LSD-t test, P<0.01) (Fig. 6A and 6B). Furthermore, there was no difference in the percentage of IL-17A-producing Th cells between the groups stimulated by induced sclerotic cells in the presence or absence of 5 µg/mL Dectin-1 blocking mAb (Fig. 6A and 6B). Notably, the percentage of IL-17A-producing Th cells was significantly higher in the group stimulated by induced sclerotic cells which was pretreated with chitinase than that in the group stimulated by induced sclerotic cells without chitinase treatment or by saprophytic *F. pedrosoi* (LSD-t test, P<0.01) (Fig. 6A and 6B). However, in the presence of Dectin-1 blockage, a significantly decreased percentage of IL-17A-producing Th cells was observed in the group stimulated by induced sclerotic cells with chitinase pretreatment (LSD-t test, P<0.01) (Fig. 6A and 6B).

## Discussion

Previous studies have shown that recruitment of CD4+, although not CD8+, T cell-mediated immune response contributes to the host defense against experimental chromoblastomycosis [3], [9]. In addition, the change of in situ Th2 cytokine pattern into Th1 pattern was observed in the infected footpad due to *F. pedrosoi* in a self-healing murine model [28]. In the present study, we have also shown that it is the T lymphocytes that play a key role in the regression of localized footpad infection due to *F. pedrosoi* using an immunocompetent as well as athymic (nu/nu) BALB/c murine model.

It is well established that the IL-17/IL-8 axis-dependent regulation of neutrophil infiltration and associated MPO expression play important roles in ROS-mediated killing of microbes including fungal pathogens [12], [13], [19], [29]. In addition, the destroyed sclerotic cells were observed in the neutrophilic center of the abscess in the experimental murine model of chromoblastomycosis due to *F. pedrosoi* [23]. Here we showed that despite persistent expression of the Th17-defining transcription factor Rorγt with obvious neutrophil infiltration in the infected footpad of self-healing model, a suppressed expression of Rorγt with decreased recruitment of neutrophils occurred in the spleen of animals that were chronically infected by the i.p. route. Furthermore, we demonstrated that Dectin-1-expressing histocytes and neutrophils participated in the enclosure of sclerotic cells.

Notably, in contrast to the inhibited expression of Rorγt in chronically infected murine spleen mentioned above, an effective Th17 immune response in splenocytes from chronic infection models was triggered by in vitro stimulation with saprophytic *F. pedrosoi*. Results from abrogation tests further suggested that Dectin-1 partly mediated the development of Th17 cells.

Whereas Dectin-1 has the capacity to specifically bind to β-glucan moieties on the cell wall of saprophytic *F. pedrosoi* as previously noted [3], our findings suggest that the capacity of Dectin-1 to bind to induced sclerotic cells decreased significantly. Chitin moieties participate in the septum formation of fungal cells, and multi-septate or muriform structure is one of the distinctive characteristics of sclerotic cells [2], [23], [30], [31]. In the present study, we detected the distribution of chitin moiety on the sclerotic cells, and demonstrated that it is the chitin moiety, but not the mannose component, that interferes with the immunorecognition of β-glucans on in vitro-induced sclerotic cells by human/murine-derived Dectin-1. Intriguingly, Wagener and colleagues show that the chitin particles with different size extracted from the yeast cells have the potential to stimulate the secretion of IL-10 or TNF-a from innate immune cells in a manner dependent on interaction with MR [32]. In our study, whereas the specific binding of WGA to chitin moiety was observed on the induced sclerotic cells, the binding site of human or murine-derived MR was mainly restricted to the hyphal elements, but not the transformed sclerotic cells or chlamydospores. We infer that there might exist some differences in conformational structure between natural chitin moieties on the induced sclerotic cells of *F. pedrosoi* and the chitin particles purified from yeast cells.

More importantly, we further demonstrated that the chitin moieties of in vitro-induced sclerotic cells contributed to the inhibition of Dectin-1-mediated Th17 development, although the percentage of Dectin-1+CD11c+ cells in the CD11c-expressing splenocytes, which were considered to include the vast majority of dentritic cells in the murine spleen [33], remained almost unchanged during an i.p. infection with *F. pedrosoi.*

With the transformation of *F. pedrosoi* mycelium into the parasitic phase- the sclerotic cells- in the spleen of chronically infected BALB/c model, we believe that the masking of β-glucans by chitin moiety may lead to an impaired Dectin-1-mediated Th17 development, and therefore, attenuate the recruitment of MPO-expressing neutrophils into the infectious foci. That hydrogen peroxide, one major source of ROS, has the ability to destroy the in vitro-induced sclerotic cells, suggests that the decreased infiltration of neutrophils into the infectious foci and inefficient MPO-catalyzed oxidation may contribute to the persistence of sclerotic cells and chronicity of infection due to *F. pedrosoi*. This was supported by the fact that the expression of MPO was not detected in the polynuclear giant cells- the hallmark of chronic infection including chromoblastomycosis- in which the sclerotic cells were separated from the infiltrated neutrophils [1], [34].

It should be mentioned that Sousa et al. demonstrated that Dectin-1 plays only a minor role during *F. pedrosoi*-infection in vivo [3]. In the present study, we have noted that the masking effect of chitin-like component mentioned above may be one factor contributing to the failure of Dectin-1-mediated immunity against experimental *F. pedrosoi*-infection.

Although the inoculated saprophytic *F. pedrosoi* turned into the sclerotic cells in the infected footpad of self-healing BALB/c model, it is possible that the persistent neutrophil infiltration and relatively higher ROS production may contribute to the destruction of chitin on the cell wall of parasitic sclerotic cells, resulting in re-exposure of masked β-glucan moieties. We are in the process of investigating whether this will recover the Dectin-1-mediated Th17 response, and facilitate the self-healing of local infected footpad.

Finally, we noted that chitin digestion contributed to the Th17 development on condition that the expression of Dectin-1 on the CD11c+ splenocytes was completely abrogated, which suggested that the chitin moiety may mask some Th17-stimulating components other than β-glucans on the cell wall of in vitro-induced sclerotic cells.

In summary, our work presents data to show that the masking effect of chitin moieties contributes to an inhibited Th17 development to some extent. The findings extend our understanding of the pathogenesis of chromoblastomycosis due to *F. pedrosoi*.
