# Correction: A Chitin-Like Component on Sclerotic Cells of *Fonsecaea pedrosoi* Inhibits Dectin-1-Mediated Murine Th17 Development by Masking β-Glucans

**DOI:** 10.1371/journal.pone.0119244

**Published:** 2015-03-18

**Authors:** The PLOS ONE Staff

The catalog number for the anti-mouse Dectin-1 blocking mAb is listed incorrectly as “FAB17561, R&D systems” in the “Cell culture, Dectin-1 blockage, and stimulation with *F*.*pedrosoi*” sub-section of the “Materials and Methods” section. The correct catalog number is “MAB17561, R&D systems."
